# Facile Construction of 2D/2D ZnIn_2_S_4_-Based Bifunctional Photocatalysts for H_2_ Production and Simultaneous Degradation of Rhodamine B and Tetracycline

**DOI:** 10.3390/nano13162315

**Published:** 2023-08-12

**Authors:** Yue Chen, Liezhen Zhu, Youliang Shen, Jing Liu, Jiangbo Xi, Lingfang Qiu, Xun Xu, Dandan Men, Ping Li, Shuwang Duo

**Affiliations:** 1 Jiangxi Key Laboratory of Surface Engineering, School of Materials and Energy, Jiangxi Science and Technology Normal University, Nanchang 330013, China; 15755612430@163.com (Y.C.); m13177845737@163.com (L.Z.); 1020101021@jxstnu.edu.cn (Y.S.); mallliu@163.com (J.L.); qlf1108@163.com (L.Q.); rozen121@163.com (X.X.); mendandan1999@126.com (D.M.); 2 Key Laboratory of Novel Biomass-Based Environmental and Energy Materials in Petroleum and Chemical Industry, Key Laboratory of Green Chemical Engineering Process of Ministry of Education, Engineering Research Center of Phosphorus Resources Development and Utilization of Ministry of Education, Hubei Key Laboratory of Novel Reactor and Green Chemical Technology, School of Chemistry and Environmental Engineering, Wuhan Institute of Technology, Wuhan 430073, China; jbxi@wit.edu.cn

**Keywords:** TiO_2_/ZnIn_2_S_4_, 2D/2D heterostructures, bifunctional photocatalysts, degradation, hydrogen evolution

## Abstract

A two-dimensional/two-dimensional (2D/2D) TiO_2_/ZnIn_2_S_4_ photocatalyst was reasonably proposed and constructed by a two-step oil bath-hydrothermal method. TiO_2_ nanosheets uniformly grown on the surface of ZnIn_2_S_4_ nanosheets and a synergetic effect between the TiO_2_ and ZnIn_2_S_4_ could highly contribute to improving the specific surface area and hydrophilicity of ZnIn_2_S_4_ as well as accelerating the separation and transfer of photon-generated e^−^-h^+^ pairs, and thus enhancing the visible-light photocatalytic degradation and H_2_ evolution performance of ZnIn_2_S_4_. Rhodamine B (RhB) and tetracycline (TC) were simultaneously selected as the target pollutants for degradation in the work. The optimum photocatalytic RhB and TC degradation properties of TiO_2_/ZnIn_2_S_4_-10 wt% were almost 3.11- and 8.61-fold higher than that of pure ZnIn_2_S_4_, separately, while the highest photocatalytic hydrogen evolution rate was also observed in the presence of TiO_2_/ZnIn_2_S_4_-10wt% and 4.28-fold higher than that of ZnIn_2_S_4_. Moreover, the possible photocatalytic mechanisms for enhanced visible-light photocatalytic degradation and H_2_ evolution were investigated and proposed in detail. Our research results open an easy pathway for developing efficient bifunctional photocatalysts.

## 1. Introduction

Since the concept of sustainable development was proposed, the production of clean energy and the treatment of wastewater with persistent organic pollutants have attracted increasing attention from researchers [1,2,3,4]. Compared to conventional treatment methods, photocatalysis technology by semiconductors has some advantages of clean, easy operation and high efficiency, which is considered to be promising in the territory of alleviating energy shortages and environmental crises [5,6]. Numerous scholars have been endeavoring to probe newfashioned semiconductors photocatalysts with superior activity and good stability to achieve effective hydrogen production and pollutant degradation in the past few decades [7,8]. Among the semiconductors photocatalysts, ternary metal chalcogenide semiconductors, such as CuCo_2_S_4_, ZnIn_2_S_4_, and CaIn_2_S_4_, have obtained exceeding attention in the domain of photocatalysis research owing to the advantages of small band gaps, outstanding photoconversion capacity, and good stability [9,10,11].

ZnIn_2_S_4_, as an outstanding representative of ternary metal chalcogenides, possesses two-dimensional (2D) nanosheet morphologies, a narrow bandgap of ca. 2.4 eV, and good stability, and thus is recognized to be a suitable candidate for visible-light photocatalytic hydrogen production and pollutant degradation [12,13]. Nonetheless, pristine ZnIn_2_S_4_ tends to agglomerate and displays low separation efficiency of the photogenerated electron-hole pairs, which greatly restricts its photocatalytic property with hindering its application in the photocatalytic realm [14]. Therefore, it is urgently needed to surmount the drawbacks of pristine ZnIn_2_S_4_, and thus a series of modification strategies have been proposed. Among all of them, constructing heterojunctions with other semiconductor materials can immensely promote the separation of photogenerated electron-hole pairs, which has been demonstrated to be a productive modification strategy [15,16].

Among the semiconductor materials, TiO_2_, as a wide bandgap (E_g_~3.2 eV) semiconductor material, is widely considered as an ideal candidate to fabricate heterojunction with ZnIn_2_S_4_ due to its excellent stability, nontoxicity, and low cost [17], which has been widely applied in photocatalytic H_2_ production [18], pollution degradation [19], CO_2_ reduction [20], and organic synthesis [21]. More importantly, the energy band position of ZnIn_2_S_4_ is above that of TiO_2_ allowing for photogenerated carriers transfer between ZnIn_2_S_4_ and TiO_2_, and thus coupling ZnIn_2_S_4_ and TiO_2_ contributes to addressing the shortfalls of ZnIn_2_S_4_ [22]. So far, TiO_2_/ZnIn_2_S_4_ heterojunctions with different morphologies, such as 2D/3D TiO_2_ nanosheets/ZnIn_2_S_4_ nanostructure [23,24], 3D ZnIn_2_S_4_ nanosheets/TiO_2_ nanobelts [25], and 1D TiO_2_ nanofibers/2D ZnIn_2_S_4_ nanosheet heterostructure [26], have been successfully fabricated with significantly improving the separation efficiency and lifetime of carriers, and thus boosting the photocatalytic activity of ZnIn_2_S_4_. It is widely believed that the 2D/2D structure with close contacts has potential advantages of large specific surface area, excellent light absorption ability, and effective charge separation efficiency [27,28,29]. It was revealed that due to the 2D/2D structure, Co_3_O_4_/ZnIn_2_S_4_ and TiO_2_/g-C_3_N_4_ photocatalysts showed efficient separation of photogenerated carriers, and thus obtaining enhanced photocatalytic properties [30,31]. Therefore, it is necessary to fabricate 2D/2D TiO_2_/ZnIn_2_S_4_ nanostructures and investigate the enhanced photocatalytic activity.

Enlightened by the aforementioned studies, we attempt to design and synthesize the TiO_2_/ZnIn_2_S_4_ nanocomposites with intimate contacted 2D/2D structure by growing TiO_2_ nanosheets on the surfaces of ZnIn_2_S_4_ nanosheets. The synergistic effect between TiO_2_ and ZnIn_2_S_4_ promoted the photogenerated carriers’ separation as well as enhanced specific surface area and hydrophilicity. As a result, the as-obtained composite photocatalysts showed significantly enhanced photocatalytic H_2_ production rate and pollution removal efficiency with excellent reusability. The charge separation and transfer mechanism on the contact interface of TiO_2_ and ZnIn_2_S_4_ for the superior photocatalytic performance was analyzed in-depth. This study provides a promising path for the construction of highly efficient photocatalysts for simultaneous application in energy- and environment-related areas.

## 2. Materials and Methods

### 2.1. Chemicals

HF (40% aqueous solution), tetraisopropyl titanate (TIPT, ≥95.0%), NaOH (≥99.0%), chromic chloride (CdCl_3_, ≥99.0%), and indium chloride (InCl_3_, ≥99.9%) were purchased from Macklin Biochemical Co., Ltd. (Shanghai, China). RhB (≥99.0%), TC (≥99.0%), Thioacetamide (TAA, ≥99.0%), anhydrous ethanol (≥99.7%), hydrochloric acid (HCl, 36.5%), and zinc chloride (ZnCl_2_, ≥98.0%) were commercially available from Sinopharm Chemical Reagent Co., Ltd. (Shanghai, China). Ethylenediamine tetraacetic acid disodium (EDTA-2Na, ≥99.5%), p-Benzoquinone (BQ, ≥99.5%), tertiary butyl alcohol (t-BuOH, ≥99.5%), and triethanolamine (TEOA, ≥98.0%) were supplied by Shanghai Zhanyun Chemical Co., Ltd. (Shanghai, China). All chemicals were utilized as received. The distilled water was obtained using Water Purification System.

### 2.2. Synthesis of ZnIn_2_S_4_ Nanosheets

ZnIn_2_S_4_ was prepared via an oil-bath process according to the former literature [12]. 272 mg of ZnCl_2_, 442 mg of InCl_3_, and 300 mg of TAA were dissolved into 50 mL of deionized water (pH = 2.5), and heated at 80 °C for 2 h. After cooling, ZnIn_2_S_4_ could be obtained by separating, washing, and drying under a vacuum at 60 °C overnight. Finally, 500 mg of ZnIn_2_S_4_ was dispersed into 100 mL of methanol with continuous ultrasound treatment. 

### 2.3. Synthesis of TiO_2_/ZnIn_2_S_4_ Nanosheets

**Figure 1:**
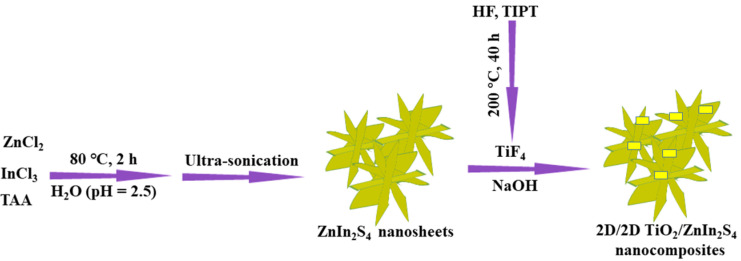
Schematic illustration for the formation process of TiO_2_/ZnIn_2_S_4_ composites.

The specific reaction process was illustrated in Figure 1. Firstly, 10 mL of HF was slowly dropped into a 100 mL of Teflon-lined autoclave reactor containing 25 mL of tetrabutyl titanate and heated at 200 °C for 40 h. After cooling, the precipitates were thoroughly separated by centrifugation and then dried under vacuum at 60 °C for overnight. Subsequently, the precipitates (3.1 mg, 15.5 mg, 18.6 mg, and 31 mg) and ZnIn_2_S_4_ dispersion liquid (19.6 mL, 18 mL, 17.6 mL, and 16 mL) were added in 0.1 M NaOH solution under stirring for 24 h, separately, then washed with deionized water till the pH = 7 and dried at 100 °C in a vacuum drying chamber. Finally, TiO_2_/ZnIn_2_S_4_ composites with different weight percent of TiO_2_ (2 wt%, 10 wt%, 12 wt% and 20 wt%) were obtained via the procedure and marked as TiO_2_/ZnIn_2_S_4_-2 wt%, TiO_2_/ZnIn_2_S_4_-10 wt%, TiO_2_/ZnIn_2_S_4_-12 wt% and TiO_2_/ZnIn_2_S_4_-20 wt%, respectively. Further, the identical method was also utilized to synthesize blank TiO_2_ in the absence of ZnIn_2_S_4_.

### 2.4. Characterization

The crystal phases were investigated via an X-ray diffractometer (XRD, XRD-6100, Shimadzu, Kyoto, Japan) using Cu-Kα radiation (λ = 1.5406 Å). The morphologies and lattice properties were analyzed by scanning electron microscopy (SEM, Sigma, Carl Zeiss, Oberkochen, Germany) and transmission electron microscopy (TEM, JEM2100, JEOL, Kyoto, Japan). The specific surface areas were determined by the physical adsorption of N_2_ on a Micromeritics (ASAP 2020, Micromeritics, Atlanta, GA, USA) using the Brunauer–Emmett–Teller (BET) equation. The chemical state was analyzed by X-ray photoelectron spectroscopy (XPS, Thermo Scientific K-Alpha, Thermo Fisher, Waltham, MA, USA). The light absorption as well as charge separation and transfer efficiency were studied by ultraviolet-visible diffuse reflection spectroscopy (UV–vis DRS, Lambda 750, PerkinElmer, Waltham, USA), photoluminescence spectroscopy (PL, ZolixLSP-X500A, Zolix, Beijing, China), fluorescence lifetime spectrophotometer (C11367, Quantaurus-Tau, Hamamatsu, Japan), and three-electrode photoelectrochemical cell system (CHI660E, Chenghua, Shanghai, China). The water contact angles were measured by a contact angle meter (HARKE-SPCA, HARKE, Beijing, China). TOC analyzer (TOC-2000, Metash, Shanghai, China) was utilized to investigate the total organic carbon (TOC) of the residual solution. 

### 2.5. Density Functional Theory (DFT) Calculation

To calculate the band gaps and work functions of TiO_2_ and ZnIn_2_S_4_, the model of TiO_2_ (1 × 2 × 1 supercell) and ZnIn_2_S_4_ (1 × 1 × 2 supercell) were first built and given in Figure S1. Subsequently, the calculations were performed by utilizing the Vienna ab initio Simulation Package (VASP), which implements the DFT with a generalized gradient approximation (GGA) and super-soft pseudopotential method. Calculations were carried out by utilizing the Predew–Burke–Ernzerhof (PBE) scheme. The electron wave functions were described by the projector augmented wave (PAW) method with a cutoff energy of 300 eV and a K-point of 2 × 3 × 2.

### 2.6. Photocatalytic Hydrogen Generation

The photocatalytic hydrogen experiments were conducted in an automatic online gas analysis system (Labsolar-6A, Perfectlight, Beijing, China). A Xenon lamp of 300 W (PLS-SXE 300C, λ > 420 nm) was employed to supply the visible-light source. During the process, 20 mg of as-fabricated photocatalysts were added into a 60 mL of mixed solution (50 mL deionized water and 10 mL of TEOA) without adding H_2_PtCl_6_, and then the reaction container was installed into the photocatalytic reaction instrument and the distance between the light source and the solution was about 16 cm. Ahead of starting the reaction, the entire installation was vacuumed to remove the air until the system pressure was beneath 1.0 Kpa. Then, turned on the light source and operated the program, automatic sampling every 60 min. In the whole reaction process, the temperature of circulating cooling water was always controlled at about 5 °C. Finally, the generated blended gas was transferred to gas chromatography (GC9790) equipped with a TCD detector (LabSolar-IIIAG, Perfectlight, Beijing, China) to further detect and calculate the production of hydrogen. To investigate the reusability of the binary heterostructure, recycled hydrogen production was carried out four times using TiO_2_/ZnIn_2_S_4_-10 wt% as the photocatalyst. The apparent quantum efficiencies (AQE) for H_2_ evolution of λ = 400, 420, and 500 nm were determined in a 75 mL Pyrex glass reactor. The apparent quantum efficiency (AQE) could be determined using the following equation:

$$\mathrm{AQE}=\frac{2\times \mathrm{the}\;\mathrm{number}\;\mathrm{of}\;H_{2}\;\mathrm{evolved}\;\mathrm{molecules}}{\mathrm{the}\;\mathrm{number}\;\mathrm{of}\;\mathrm{incident}\;\mathrm{photons}}\times 100\%$$

### 2.7. Photodegradation Activity Evaluation

The visible-light photocatalytic degradation activity was evaluated by the degradation of fresh TC (10 mg/L) and RhB (30 mg/L) solution. The light source was provided by 500 W Xenon light (PLS-SXE 500C) equipped with a UV cutoff filter (λ > 420 nm). Firstly, 10 mg of photocatalyst was dispersed into the 50 mL of TC solution and 50 mL of RhB solution, respectively. Then, the above-mentioned solution was transferred to the photocatalytic reaction apparatus (XPA-7) and kept stirring in the dark for 60 min to obtain an adsorption-desorption equilibrium. After turning on the Xenon light, the photocatalytic degradation reaction was starting. At a given interval, a 4 mL aliquot of mixture was taken out utilizing a syringe with a needle and then filtrated using a 0.22 μm Millipore filter to obtain the residual solution, the concentration of which at the maximum absorption wavelength (355 nm for TC and 554 nm for RhB) was monitored using a PerkinElmer UV–vis spectrophotometer (Lambda 35). Moreover, the recycled photodegradation experiments were carried out four times using TiO_2_/ZnIn_2_S_4_-10 wt% as the photocatalyst at the same condition. Once the degradation experiment was over, the remaining sample in the beaker was immediately recycled by separation, washing, and drying for the next cyclic degradation experiment. The degradation efficiency (De %) was calculated by the equation:

$$\mathrm{De}\;\%=\frac{C_{0}-C_{t}}{C_{0}}\times 100\%$$

In the formula, C_0_ and C_t_ denote the concentrations at the initial time and after each stage of degradation, separately. As for the trapping experiments, equal amounts (1.0 mM) of scavengers were added to the TC solution to capture active radicals.

## 3. Results and Discussions

**Figure 2:**
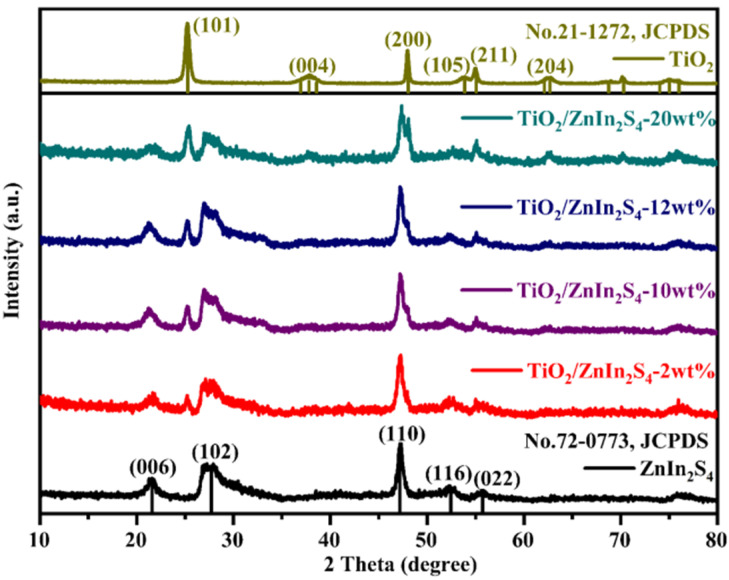
XRD patterns of the as-prepared ZnIn_2_S_4_, TiO_2_, and TiO_2_/ZnIn_2_S_4_ composites.

The text continues here. The XRD patterns were used to recognize the phase composition and the structure of samples, and the results are exhibited in Figure 2. The diffraction peaks observed at 21.6°, 27.7°, 47.2°, 52.7°, and 55.6° can be well attributed to the (006), (102), (110), (116), and (022) crystal planes of hexagonal ZnIn_2_S_4_ (JCPDS No. 72-0773) [32]. For pure TiO_2_, a set of diffraction peaks located at 25.3° (101), 37.8° (004), 48.1° (200), 54.1° (105), 55.1° (211) and 62.8° (204) are consistent with the anatase TiO_2_ (JCPDS No. 21-1272) [33]. The diffraction peaks of ZnIn_2_S_4_ and TiO_2_ can be seen simultaneously in the XRD patterns of TiO_2_/ZnIn_2_S_4_ composites, the characteristic peak intensities of TiO_2_ generally increased, while the diffraction peaks of ZnIn_2_S_4_ gradually decrease with the increased content of TiO_2_. Further, no other peaks of the third (impurity) phase are detected in all XRD patterns, implying the successful construction of the TiO_2_/ZnIn_2_S_4_ composites.

**Figure 3:**
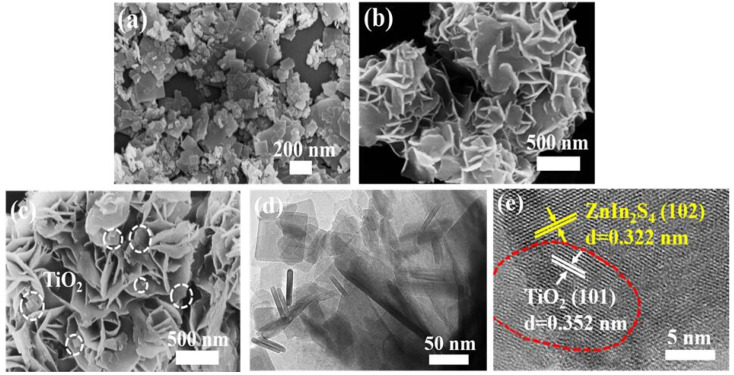
SEM images of TiO_2_ (**a**), ZnIn_2_S_4_ (**b**), and TiO_2_/ZnIn_2_S_4_-10wt% (**c**); TEM image (**d**) and HR-TEM image (**e**) of TiO_2_/ZnIn_2_S_4_-10 wt%.

SEM and TEM were utilized to observe the morphology of the catalysts. As displayed in Figure 3a, pristine TiO_2_ showed a 2D nanosheet structure with different sizes. As for pure ZnIn_2_S_4_, a nanoflower-like structure assembled by the large number of nanosheets can be seen in Figure 3b. After coupling ZnIn_2_S_4_ with TiO_2_, SEM (Figure 3c) and TEM (Figure 3d) images indicate TiO_2_ nanosheets grow on the surface of ZnIn_2_S_4_ nanosheets, forming an intimate 2D/2D contact interface between ZnIn_2_S_4_ and TiO_2_. The elemental distribution in the TiO_2_/ZnIn_2_S_4_ composite was analyzed using SEM-energy-dispersive X-ray spectroscopy (Figure S3), and the result confirmed the homogeneous coexistence of Ti, O, Zn, In, and S elements. Moreover, high-resolution TEM analysis was conducted to investigate the microstructure information of the TiO_2_/ZnIn_2_S_4_-10 wt% composite, and the result is depicted in Figure 3e, the lattice fringes with d spacings of 0.352 and 0.322 nm can be seen, which can be assigned to TiO_2_ (101) and ZnIn_2_S_4_ (102) facets, separately [34,35]. The above results further indicate the successful formation of the TiO_2_/ZnIn_2_S_4_ hybrid.

**Figure 4:**
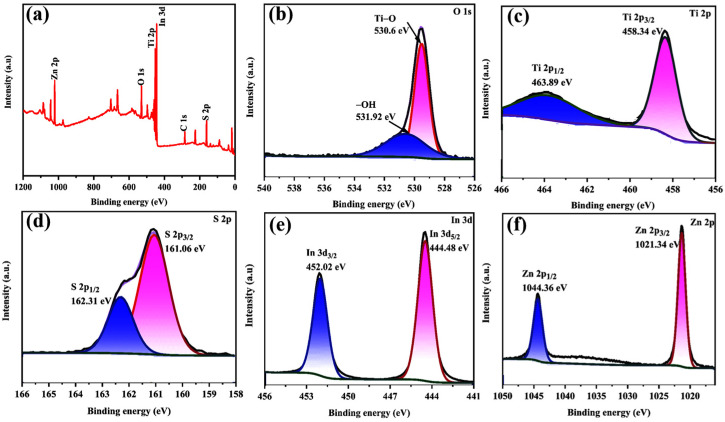
XPS spectra of as-synthesized TiO_2_/ZnIn_2_S_4_-10 wt% heterostructure: (**a**) survey scan, (**b**) O 1s, (**c**) Ti 2p, (**d**) S 2p, (**e**) In 3d, and (**f**) Zn 2p.

XPS was explored to be aware of the surface element composition and chemical state of the TiO_2_/ZnIn_2_S_4_-10 wt% composite. As shown in Figure 4a, the XPS survey spectrum of TiO_2_/ZnIn_2_S_4_ reveals the coexistence of Ti, O, S, Zn, and In elements, which is in keeping with the EDS test results. Figure 4b presents the XPS spectrum of O 1s, two characteristic peaks located at 530.6 and 531.92 eV can be attributed to the Ti-O bond and the –OH group, respectively [36]. The high-resolution XPS spectra of Ti 2p showed two characteristic peaks located at 458.34 and 463.89 eV (Figure 4c), assigning to Ti 2p_3/2_ and Ti 2p_1/2_, separately [37]. In the high-resolution S 2p spectrum (Figure 4d), the binding energies of 161.06 and 162.31 eV can be assigned to the S 2p_3/2_ and S 2p_1/2_, respectively, suggesting the occurrence of S^2−^ [38]. In Figure 4e, the peaks centered at 444.30 and 452.40 eV are ascribed to In 3d_5/2_ and In 3d_3/2_, assigning to In^3+^ binding state [39]. As for Zn 2p (Figure 4f), the peaks centered at 1021.34 and 1044.36 eV ascribed to 2p_3/2_ and 2p_1/2_, respectively, which proves the existence of Zn^2+^ [40].

**Figure 5:**
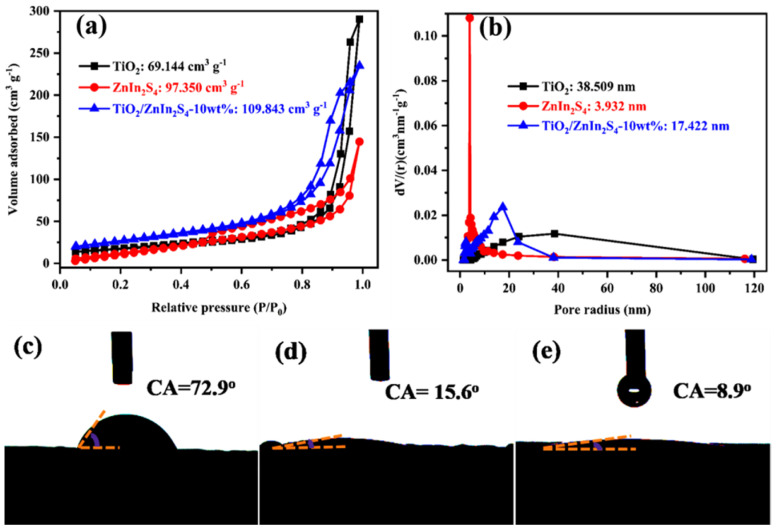
Nitrogen adsorption-desorption isotherms (**a**) and the corresponding pore size distribution plots (**b**) of bare TiO_2_, ZnIn_2_S_4_, and TiO_2_/ZnIn_2_S_4_-10 wt% samples; Water contact angles of ZnIn_2_S_4_ (**c**), TiO_2_/ZnIn_2_S_4_-10 wt% (**d**), and TiO_2_ (**e**).

The specific surface area and water contact angles were measured to investigate the adsorption performance of photocatalysts, and the results are displayed in Figure 5. The nitrogen adsorption-desorption isotherms of TiO_2_, ZnIn_2_S_4_, and TiO_2_/ZnIn_2_S_4_-10 wt% showed type IV isotherms with the hysteresis loop of mesoporous structures (Figure 5a), the specific surface area of TiO_2_/ZnIn_2_S_4_-10 wt% was larger than that of TiO_2_ and ZnIn_2_S_4_. It can be seen that the average pore sizes are between 2 and 50 nm (Figure 5b), which further confirmed the formation of a mesoporous structure. Meanwhile, water contact angles of TiO_2_, TiO_2_/ZnIn_2_S_4_, and ZnIn_2_S_4_ were also measured to analyze the hydrophilicity and hydrophobicity of prepared materials. It can be observed from Figure 5c–e that contact angles of ZnIn_2_S_4_, TiO_2_/ZnIn_2_S_4_-10 wt% and TiO_2_ were, respectively, 72.9°, 15.6°, and 8.9°, manifesting the hydrophilicity of ZnIn_2_S_4_ can be improved by coupling with TiO_2_. These results illustrated that interface contact exists between pollutants and the photocatalysts owing to the enhanced specific surface area and hydrophilicity, and thus it is expected to obtain excellent photocatalytic performance.

**Figure 6:**
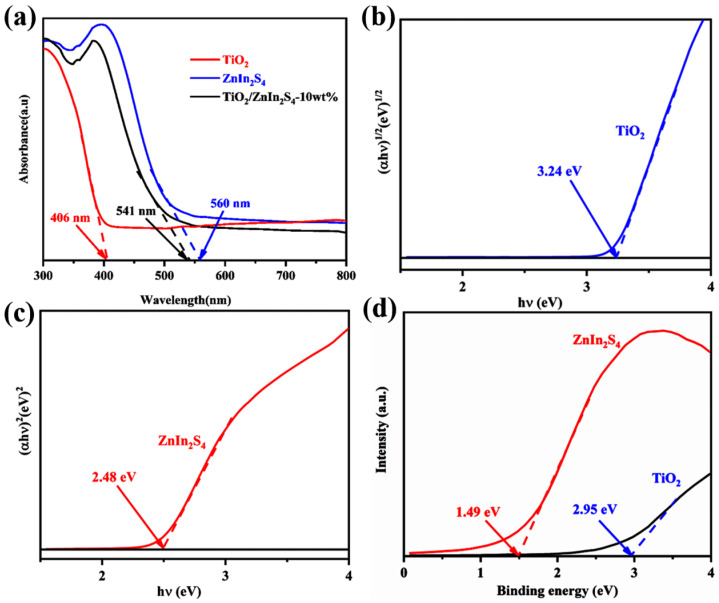
(**a**) UV–vis DRS of TiO_2_, ZnIn_2_S_4_ and TiO_2_/ZnIn_2_S_4_-10 wt%; energy bandgap of (**b**) TiO_2_ and (**c**) ZnIn_2_S_4_; (**d**) VB-XPS of TiO_2_ and ZnIn_2_S_4_.

The photoabsorptive behavior of TiO_2_, ZnIn_2_S_4_, and TiO_2_/ZnIn_2_S_4_-10 wt% was detected via UV–vis DRS spectra as shown in Figure 6a, the absorption wavelength of pristine ZnIn_2_S_4_ with steep edge was at approximate 560 nm in the visible-light areas, which presented favorable absorption capacity both in the visible and UV light, while the absorption edge of absolute TiO_2_ was located in about 406 nm. The photoabsorption ability of TiO_2_/ZnIn_2_S_4_-10 wt% exhibits a very close absorption profile with ZnIn_2_S_4_ with slightly diminished absorption and blue-shifted absorption edge, suggesting the introduction of TiO_2_ has a slight influence on the light absorption property of ZnIn_2_S_4_. As depicted in Figure 6b,c, the band gaps of TiO_2_ and ZnIn_2_S_4_ were calculated as 3.24 and 2.48 eV based on the equation: (αhν)^1/n^ = A(hν−E_g_) [41], which were roughly matched with the results of DFT calculation (Figure S3). Valence band XPS (VB-XPS) of TiO_2_ and ZnIn_2_S_4_ were also conducted to further understand the band structure of TiO_2_ and ZnIn_2_S_4_. As demonstrated in Figure 6d, the E_VB-XPS_ values of pure TiO_2_ and ZnIn_2_S_4_ were 2.95 and 1.49 eV, respectively. Therefore, the VB potentials of the normal hydrogen electrode (E_VB–NHE_, pH = 7) of TiO_2_ and ZnIn_2_S_4_ were determined to be 2.71 and 1.25 eV based on the E_VB vs. NHE_ = φ + E_VB-XPS_ − 4.44, where φ is the work function (4.2 eV) of the XPS analyzer [42], while the E_CB vs. NHE_ values of TiO_2_ and ZnIn_2_S_4_ could be computed as −0.53 and −1.23 eV using the equation: E_CB_ = E_VB_ − E_g_ [43]. Therefore, the overall band structure positions of TiO_2_ and ZnIn_2_S_4_ can be obtained and shown in Figure S4.

**Figure 7:**
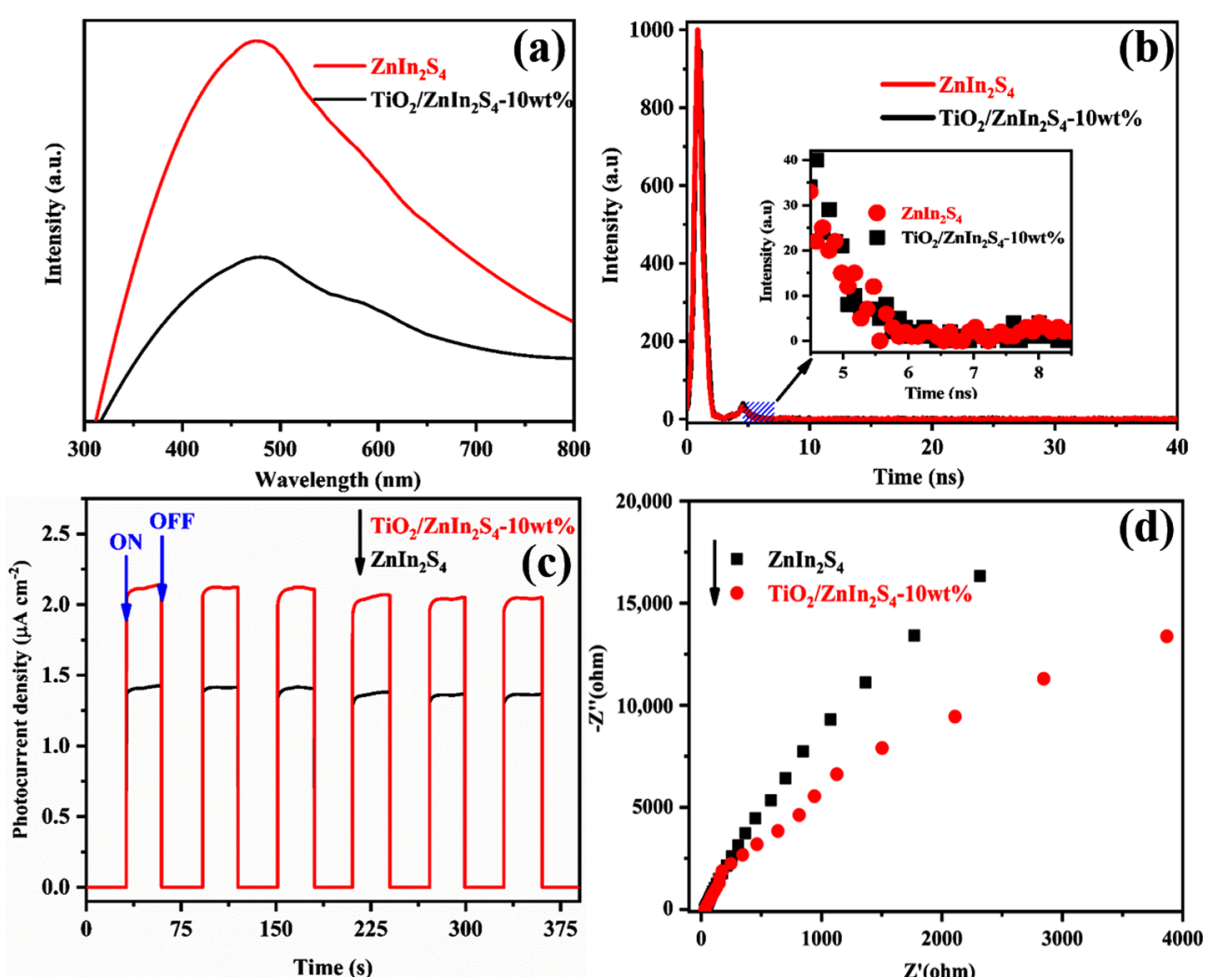
PL spectra (**a**), TRPL curves (**b**), transient photocurrent responses (**c**), and EIS plots (**d**) over bare ZnIn_2_S_4_ and TiO_2_/ZnIn_2_S_4_-10 wt% samples.

To uncover the positive influence of constructing heterojunction on the catalytic performances of ZnIn_2_S_4_, the separation and migration behaviors of photogenerated charges were deeply investigated. First, the PL spectra of ZnIn_2_S_4_ and TiO_2_/ZnIn_2_S_4_-10 wt% were measured to monitor the recombination process of photoinduced charge carriers. Generally, the dramatically reduced PL intensity is regarded as a signal of effective charge separation [44]. As shown in Figure 7a, TiO_2_/ZnIn_2_S_4_-10 wt% exhibited a lower PL intensity than ZnIn_2_S_4_, suggesting a higher separation efficiency of photogenerated carriers in TiO_2_/ZnIn_2_S_4_-10 wt% composite. The time-resolved photoluminescence (TRPL) spectra were also acquired to investigate the detailed information about the decay behavior of photogenerated carriers, and the results are shown in Figure 7b. The average fluorescence lifetime (τ_avg_) of TiO_2_/ZnIn_2_S_4_-10 wt% (581 ps) was longer than pristine ZnIn_2_S_4_ (543 ps), implying that the coupling ZnIn_2_S_4_ with TiO_2_ can helpfully prevent the recombination of photoinduced carriers and obtain a longer fluorescence lifetime. To further clarify the enhanced photogenerated charge transfer and separation efficiency, the photoelectrochemical performance was characterized and analyzed by transient photocurrent responses and EIS tests. As demonstrated in Figure 7c, the TiO_2_/ZnIn_2_S_4_-10 wt% showed a higher photocurrent density than ZnIn_2_S_4_, and the average photocurrent density of TiO_2_/ZnIn_2_S_4_-10 wt% was raised to be 2.11 mA·cm^−2^, approximately 1.5-fold larger than that of pristine ZnIn_2_S_4_ (1.39 mA·cm^−2^), implying that the construction of TiO_2_/ZnIn_2_S_4_ composite can promote the photoexcited charge carrier transfer. Furthermore, the EIS plot of TiO_2_/ZnIn_2_S_4_-10 wt% composite exhibited a smaller semicircle than pristine ZnIn_2_S_4_, as observed in Figure 7d, manifesting a lesser electric resistance and more efficient charge transfer process existing in the TiO_2_/ZnIn_2_S_4_-10 wt% composite. These optical and photoelectrochemical properties demonstrated that the formation of TiO_2_/ZnIn_2_S_4_ heterojunction was capable of elevating the separation and transfer efficiency of photogenerated carriers, thus obtaining the enhanced photocatalytic performance.

**Figure 8:**
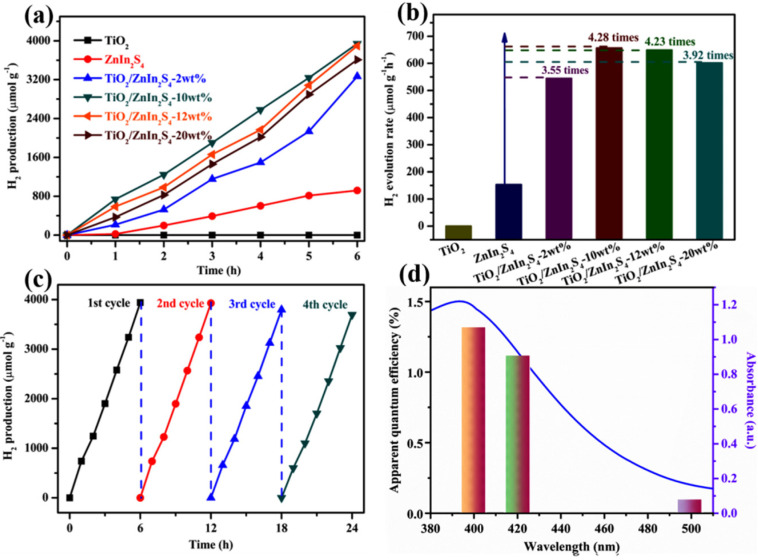
(**a**) Photocatalytic HER activity (**a**) and H_2_ evolution rates (**b**) of all samples. (**c**) Cycling experiments of TiO_2_/ZnIn_2_S_4_-10 wt%. (**d**) AQEs and DRS spectrum of TiO_2_/ZnIn_2_S_4_-10 wt%.

The visible-light photocatalytic H_2_ generation activities of TiO_2_, ZnIn_2_S_4_, and TiO_2_/ZnIn_2_S_4_ composites were evaluated in the presence of TEOA sacrificial reagent. As shown in Figure 8a, pure ZnIn_2_S_4_ possessed a low photocatalytic performance due to the high recombination rate of photoexcited charge carriers and photocorrosion, while pristine TiO_2_ had almost no catalytic activity, which could be attributed to its wide bandgap [45]. Notably, the photoactivity of ZnIn_2_S_4_ was gradually improved along with the introduction of TiO_2_. Among all composites, the TiO_2_/ZnIn_2_S_4_-10 wt% composite showed the optimal H_2_ rate of 650 μmol/h/g, which was 4.28-fold higher than that of pristine ZnIn_2_S_4_ (Figure 8b). The recycling tests were also conducted in the same reaction condition to investigate the durability performance of TiO_2_/ZnIn_2_S_4_-10 wt% photocatalyst. As is demonstrated in Figure 8c, the H_2_ production amount throughout four successive cycles barely changed. Moreover, the crystal structure and morphology of TiO_2_/ZnIn_2_S_4_-10 wt% showed no noticeable changes by comparing the XRD pattern (Figure S5a) or SEM image (Figure S5b) of a used sample with the fresh sample. These test results manifested that the TiO_2_/ZnIn_2_S_4_-10 wt% composite possesses good photocatalytic stability. To further clarify the driving force in the photocatalytic process, the AQEs of TiO_2_/ZnIn_2_S_4_-10 wt% photocatalyst at 400, 420, and 500 nm were calculated as 1.3, 1.1, and 0.1%, respectively (Figure 8d), which exhibits a similar trend with the adsorption spectrum, indicating that the H_2_ production reaction is a photocatalytic driven process.

**Figure 9:**
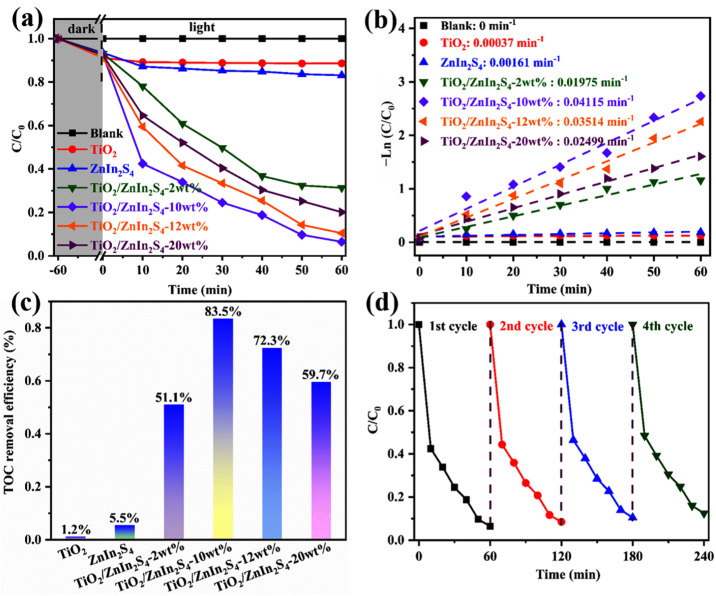
Photocatalytic degradation rate (**a**), the pseudo-first-order kinetics fitted curves (**b**), and TOC removal rate (**c**) of TC over all samples; maintenance of catalytic performance of TiO_2_/ZnIn_2_S_4_-10 wt% (**d**).

To confirm the performance multiformity of the as-prepared samples, the photocatalytic degradation capacities of all samples were also investigated by using colorless TC and colored RhB as the simulated organic pollutants. The TC and RhB photodegradation curves of TiO_2_, ZnIn_2_S_4_, and TiO_2_/ZnIn_2_S_4_ composites were illustrated in Figure 9a and Figure S6a, respectively. Almost no changes in the concentration of TC and RhB were noticed in the absence of a catalyst, suggesting that the self-degradation process could be ignored. The pure TiO_2_ displayed weak degradation activities within 60 min, while ZnIn_2_S_4_ showed high degradation activities than TiO_2_ due to a wider visible-light response range. With respect to the TiO_2_/ZnIn_2_S_4_ composites, all composites showed better photocatalytic performance than TiO_2_ and ZnIn_2_S_4_. Among them, TiO_2_/ZnIn_2_S_4_-10 wt% possessed the optimum performance, and almost 95% of TC and 93% of RhB could be degraded. The photocatalytic activity was enhanced when the mass content of TiO_2_ increased from 2% to 10%, then decreased as TiO_2_ content further increased to 12% or even more, which may be attributed to excessive TiO_2_ shielding the light absorption. To obtain the reaction rate constant “k”, the photodegradation curves were further kinetically fitted by using the pseudo-first-order equation: −ln (C/C_0_) = kt, the results were displayed in Figure 9b and Figure S6b, separately. The k value of TiO_2_/ZnIn_2_S_4_-10 wt% composite was highest compared with other samples and was up to 0.04115 min^−1^ for TC and 0.04168 min^−1^ for RhB, which was almost 111 and 190 fold that of pure TiO_2_, and 26 and 6.65 fold that of individual ZnIn_2_S_4_. Meanwhile, the mineralization capacities of all kinds of photocatalysts were investigated by TOC measurement. As demonstrated in Figure 9c and Figure S6c, the TOC removal efficiencies of TiO_2_/ZnIn_2_S_4_ composites distinctly overtopped TiO_2_ and ZnIn_2_S_4_ under the irradiation of visible light. Among them, the TiO_2_/ZnIn_2_S_4_-10 wt% composite showed the highest TOC removal efficiency (83.5% for TC and 85.6 for RhB), which formed the correspondence with its doughty photocatalytic degradation abilities, and confirmed that the TiO_2_/ZnIn_2_S_4_ had high mineralization capacities. To determine the reusability of TiO_2_/ZnIn_2_S_4_-10 wt% in the photocatalytic process, the photocatalytic cycle experiments were performed to investigate the reusable performance. As shown in Figure 9d and Figure S6d, the photodegradation efficiency scarcely had changed after undergoing four consecutive cycles. In addition, the XRD pattern (Figure S7a,c) and SEM image (Figure S7b,d) of TiO_2_/ZnIn_2_S_4_-10 wt% illuminated the crystal structure and morphology of TiO_2_/ZnIn_2_S_4_-10 wt% before and after photodegradation cycling remained unchanged. The results demonstrated the splendid degradation stability of TiO_2_/ZnIn_2_S_4_-10 wt% during the photocatalytic process.

**Figure 10:**
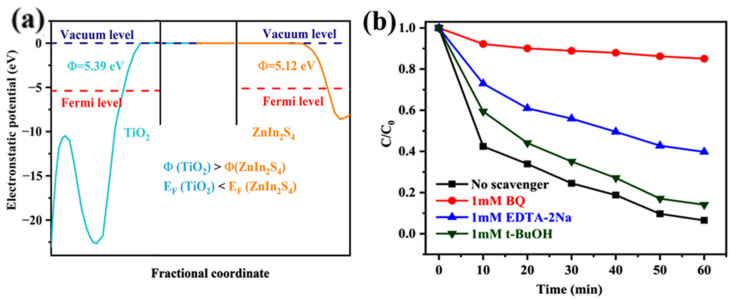
(**a**) Work functions of the TiO_2_ (001) and ZnIn_2_S_4_ (001) surfaces; (**b**) photocatalytic degradation performance of TC on TiO_2_/ZnIn_2_S_4_-10 wt% with various scavengers.

The work functions (Φ) were calculated to investigate the route of charge transfer at the contact interface of ZnIn_2_S_4_ and TiO_2_, and the results are given in Figure 10a. It was observed that the Φ of ZnIn_2_S_4_ is lower than that of TiO_2_, and thus the photoinduced electrons could transfer from ZnIn_2_S_4_ to TiO_2_ when ZnIn_2_S_4_ and TiO_2_ came in contact to construct a heterojunction. Subsequently, EDTA-2Na, t-BuOH, and BQ were selected in sequence as the scavengers of h^+^, ^•^OH, and ^•^O_2_^−^ to further identify the roles of active species during the photodegradation process. As recorded in Figure 10b, varying degrees of photocatalytic activity suppression were observed after sacrificial agents were added with an order BQ > EDTA-2Na > t-BuOH, indicating ^•^O_2_^−^ and h^+^ are main and secondary active substances, separately, while ^•^OH has minimal impact on the photocatalytic reactions. 

**Figure 11:**
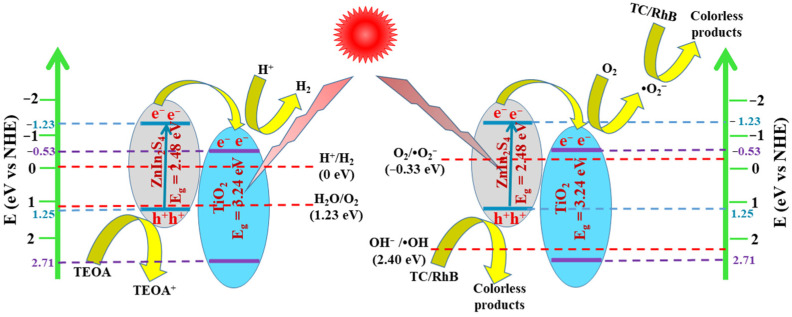
Proposed mechanisms for the photocatalytic reaction on the TiO_2_/ZnIn_2_S_4_ composite.

The possible mechanisms for boosting photocatalytic pollutant degradation and H_2_ production performances of TiO_2_/ZnIn_2_S_4_ composites were proposed and illustrated in Figure 11 based on the aforementioned discussion. When TiO_2_ nanosheets grew on the surface of ZnIn_2_S_4_ nanosheets, a closed contact interface was formed between ZnIn_2_S_4_ and TiO_2_. Under visible-light irradiation, TiO_2_ could not absorb visible light due to its large band gap energy, while the electrons on the VB of ZnIn_2_S_4_ could be easily excited to its CB and generate electron-hole pairs because of the small band gap energy. According to the DFT calculated results of work functions, the electrons would transfer from the CB of ZnIn_2_S_4_ to that of TiO_2_, while h^+^ left on the VB of ZnIn_2_S_4_, which leads to the spatial separation of electrons and holes with higher redox powers. For H_2_ production, the photogenerated e^−^ could easily reduce the surface-adsorbed protons to H_2_ with h^+^ being consumed by TEOA. Different from the H_2_ production process, electrons on the CB of TiO_2_ would firstly react with O_2_ to obtain ^•^O_2_^−^ in the photodegradation process. Subsequently, the h^+^ and ^•^O_2_^−^ participated in the photodegradation reaction due to their strong oxidation ability. As a result, the improved separation efficiency of photoinduced charge carriers would provide more carriers to participate in the photocatalytic reaction process to acquire enhanced photocatalytic performance.

## 4. Conclusions

In summary, a 2D/2D heterojunction consisting of ZnIn_2_S_4_ nanosheets and TiO_2_ nanosheets was fabricated by a facile two-step synthesis method for photocatalytic H_2_ evolution and pollutant degradation. The small TiO_2_ nanosheets deposited on the surface of large ZnIn_2_S_4_ nanosheets, resulting in the formation of the close 2D/2D heterointerface contact, which contributes to providing sufficient and short paths for the separation and transfer of photoinduced charge. All TiO_2_/ZnIn_2_S_4_ heterojunction photocatalysts possess higher photocatalytic activities than pure ZnIn_2_S_4_ and TiO_2_. Among them, the TiO_2_/ZnIn_2_S_4_-10 wt% photocatalyst exhibits optimal H_2_ evolution rate (650 μmol/h/g) and pollution degradation efficiencies (95% for TC and 93% for RhB) with excellent photocatalytic stability during four consecutive test cycles. The enhanced photocatalytic properties are believed to have originated from the accelerated charges separation and transfer as well as enhanced specific surface area and hydrophilicity. This work provides a practical strategy for preparing ZnIn_2_S_4_-based heterojunctions to act as highly efficient bifunctional photocatalysts for energy and environmental application.

## Data Availability

The authors confirm that the data supporting the findings of this study are available within the article. Derived data supporting the findings of this study are available on request.

## Supplementary Materials

The following supporting information can be downloaded at: https://www.mdpi.com/article/10.3390/nano13162315/s1, Figure S1: structure models; Figure S2: The elemental mapping images; Figure S3: Calculated band structures; Figure S4: The energy band structure; Figure S5: XRD patterns and SEM image in the recycled photocatalytic H_2_ development; Figure S6: Photocatalytic degradation rate, pseudo-first-order kinetics fitted curves, TOC removal rate and maintenance of catalytic performance; Figure S7: XRD patterns and SEM images in the recycling photocatalytic degradation.
