# Supplementary material for: Facile Construction of 2D/2D ZnIn2S4-Based Bifunctional Photocatalysts for H2 Production and Simultaneous Degradation of Rhodamine B and Tetracycline
Source: Nanomaterials (Basel). 2023 Aug 12;13(16):2315. doi: 10.3390/nano13162315 (PMC10459443; doi:10.3390/nano13162315)
Supplement: Supplementary file 1 [file nanomaterials-13-02315-s001.zip › nanomaterials-2550534-supplementary.pdf]

Supplementary Materials

Facile Construction of 2D/2D $ZnIn_2S_4$-Based Bifunctional Photocatalysts for H_2 Production and Simultaneous Degradation of Rhodamine B and Tetracycline

Yue Chen [1], Liezhen Zhu [1], Youliang Shen [1], Jing Liu [1], Jiangbo Xi [2], Lingfang Qiu [1], Xun Xu [1], Dandan Men [1], Ping Li [1,*] and Shuwang Duo [1,*]

[1] Jiangxi Key Laboratory of Surface Engineering, School of Materials and Energy, Jiangxi Science and Technology Normal University, Nanchang 330013, China; 15755612430@163.com (Y.C.); m13177845737@163.com (L.Z.); 1020101021@jxstnu.edu.cn (Y.S.); mallliu@163.com (J.L.); qlf1108@163.com (L.Q.); rozen121@163.com (X.X.); mendandan1999@126.com (D.M.)

[2] Key Laboratory of Novel Biomass-Based Environmental and Energy Materials in Petroleum and Chemical Industry, Key Laboratory of Green Chemical Engineering Process of Ministry of Education, Engineering Research Center of Phosphorus Resources Development and Utilization of Ministry of Education, Hubei Key Laboratory of Novel Reactor and Green Chemical Technology, School of Chemistry and Environmental Engineering, Wuhan Institute of Technology, Wuhan 430073, China; jbxi@wit.edu.cn

* Correspondence: 1020170927@jxstnu.edu.cn (P.L.); dsw@jxstnu.edu.cn (S.D.)

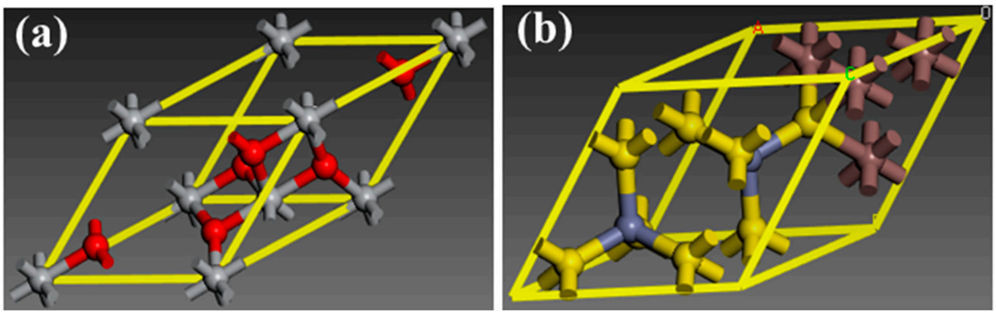

Figure S1. Structure models of TiO_2 (a) and $ZnIn_2S_4$ (b).

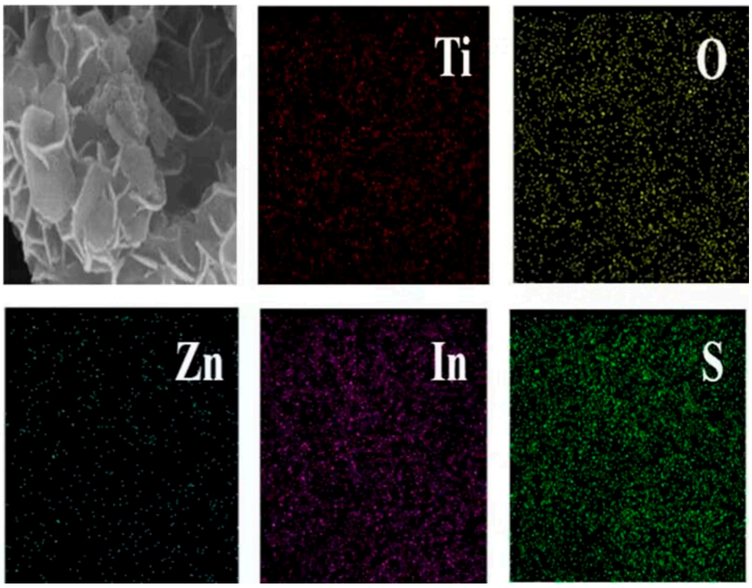

Figure S2. The elemental mapping images of $TiO_2/ZnIn_2S_4$-10 wt%.

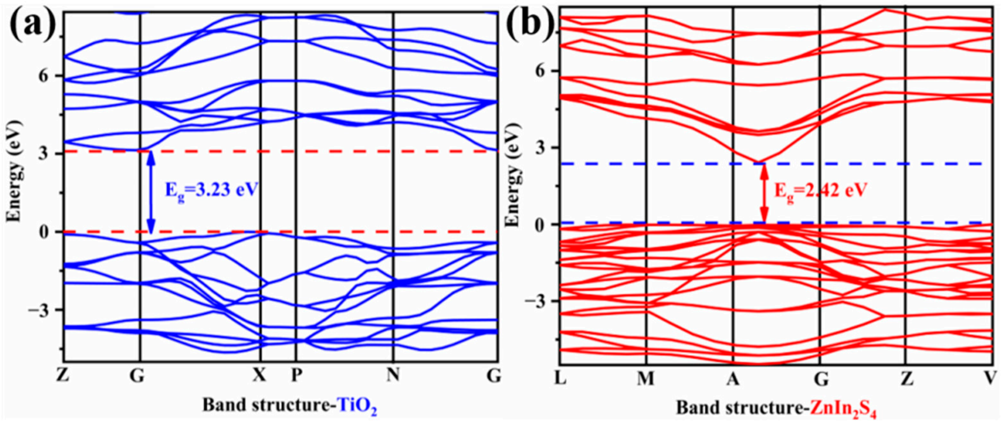

Figure S3. Calculated band structures of TiO_2 (a) and $ZnIn_2S_4$ (b).

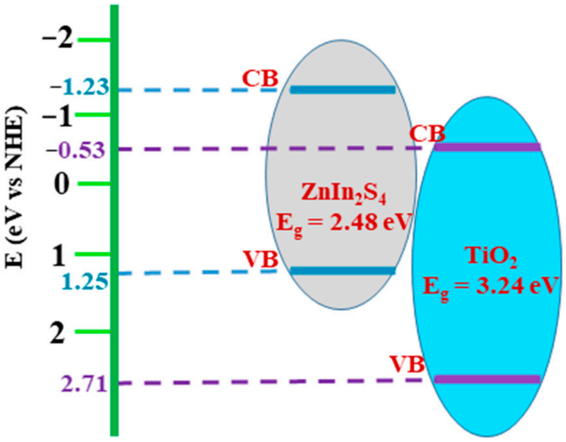

Figure S4. The energy band structure of TiO_2 and $ZnIn_2S_4$.

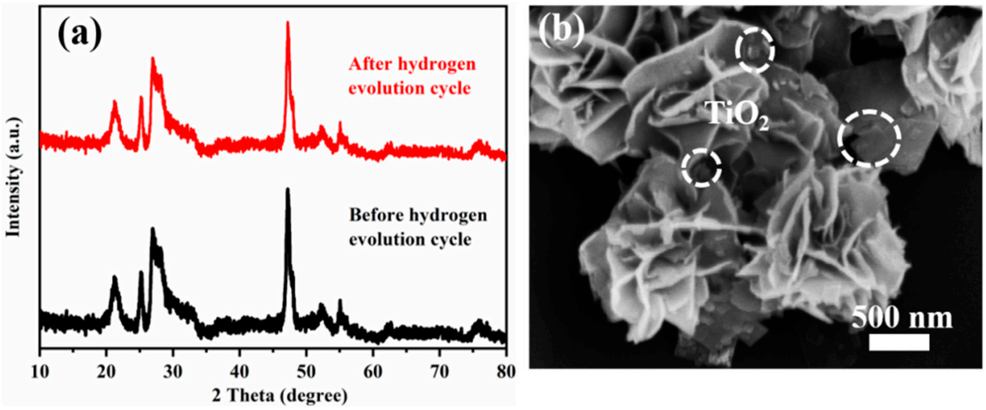

Figure S5. The XRD patterns (a) and SEM image (b) of $TiO_2/ZnIn_2S_4$-10 wt% in the recycled photocatalytic H_2 development.

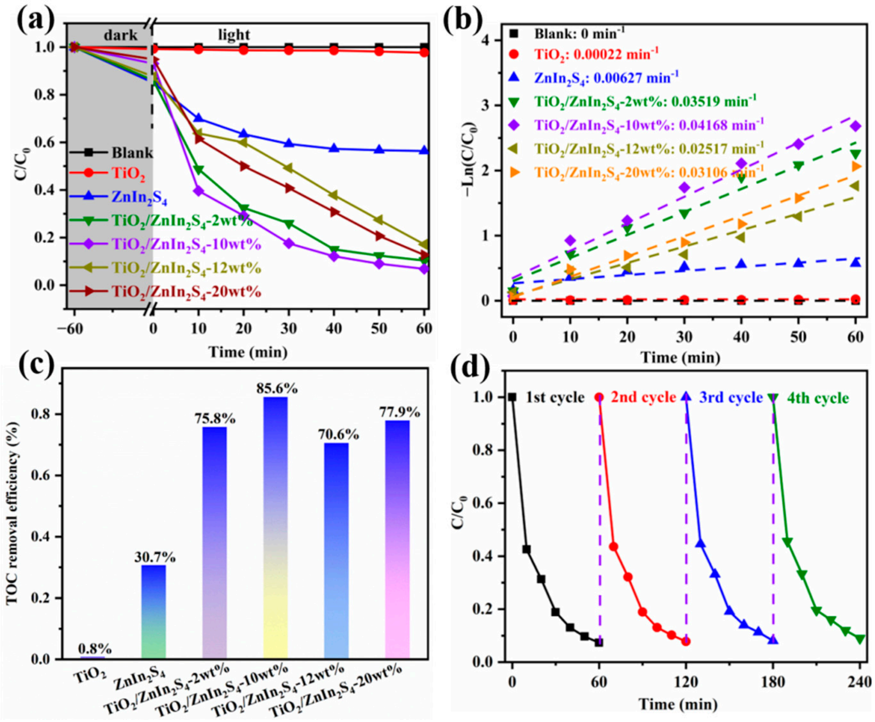

Figure S6. Photocatalytic degradation rate (a), the pseudo-first-order kinetics fitted curves (b), and TOC removal rate (c) of RhB over all samples; Maintenance of catalytic performance of $TiO_2/ZnIn_2S_4$-10 wt% (d).

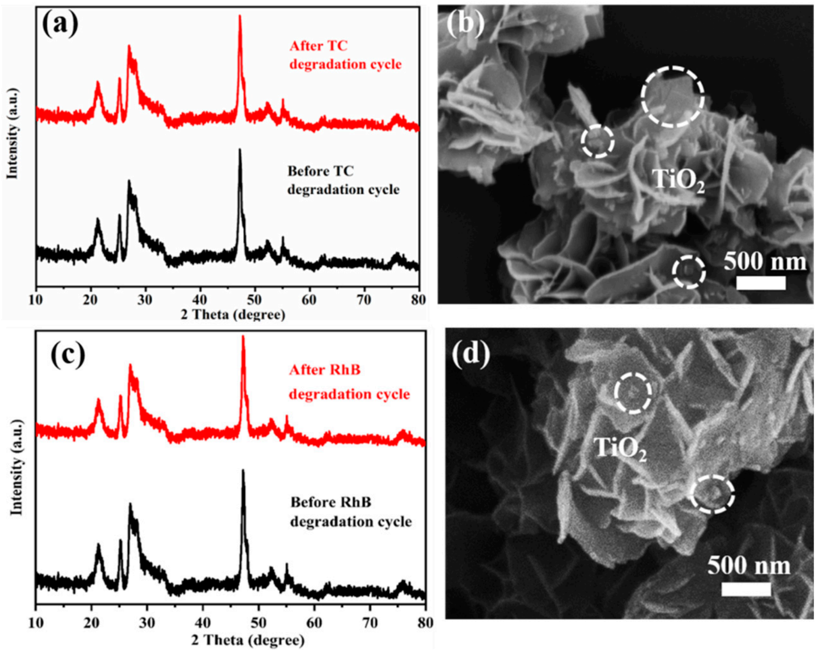

Figure S7. The XRD patterns and SEM images of $TiO_2/ZnIn_2S_4$-10 wt% in the recycling photocatalytic degradation of TC (a,b) and RhB (c,d).
